# Implementation fidelity of an intervention programme to enhance adherence to antihypertensive medication in Dutch community pharmacies

**DOI:** 10.1007/s11096-019-00845-z

**Published:** 2019-05-15

**Authors:** Danielle M. van der Laan, Marlous Langendoen-Gort, Giel Nijpels, Christel C. L. M. Boons, Petra J. M. Elders, Jacqueline G. Hugtenburg

**Affiliations:** 1 Department of Clinical Pharmacology and Pharmacy, Amsterdam Public Health Research Institute, Amsterdam UMC, Vrije Universiteit Amsterdam, De Boelelaan 1117, Amsterdam, The Netherlands; 2 Department of General Practice and Elderly Care Medicine, Amsterdam Public Health Research Institute, Amsterdam UMC, Vrije Universiteit Amsterdam, De Boelelaan 1117, Amsterdam, The Netherlands

**Keywords:** Community pharmacies, Implementation fidelity, Medication non-adherence, Patient-tailored intervention, Process evaluation, The Netherlands

## Abstract

*Background* Insight into the delivery of interventions is necessary to gain a better understanding of what caused an intervention to succeed or fail. The Cardiovascular medication non-Adherence Tailored Intervention (CATI) study failed to show effectiveness of a patient-tailored, pharmacist-led intervention programme on self-reported adherence to antihypertensive medication. *Objective* To evaluate the implementation fidelity of the CATI intervention programme. *Setting* Twenty Dutch community pharmacies. *Method* The process of a randomised controlled trial was evaluated. Both quantitative and qualitative data were collected and analysed according to Carrolls’ Conceptual Framework for Implementation Fidelity. Implementation fidelity is defined as the degree to which the intervention was implemented as intended. *Main outcome measure* Four key intervention components of the intervention programme (i.e., first consultation: barrier identification, information and advice, written summary, and follow-up consultation). *Results* For most participants the key intervention components were implemented as intended. The training of pharmacists, intensive monitoring during the study and structured and easy-to-use intervention materials facilitated the implementation of the intervention. The method to select participants for the intervention programme was considered insufficient and pharmacists questioned the eligibility of some participants because of a low degree of intake non-adherence. *Conclusion* Implementation fidelity was moderate to high for all key intervention components. Therefore, the absence of effectiveness of the CATI intervention programme on self-reported medication adherence cannot be explained by poor implementation of the intervention. However, the limited genuine eligibility of some participants resulted in a limited potential for improvement in medication adherence.

## Impacts on practice

- Pharmacist-led consultations to discuss patients’ medication adherence and barriers to adhere to medication seem feasible.
- The challenge remains to identify a patient group that is eligible for adherence enhancing interventions.
- Extensive communication skills training, easy-to-use and system integrated intervention materials and sufficient time seem necessary to implement adherence enhancing interventions in daily practice.

## Introduction

In the last few decades, multiple pharmacist-led interventions have been developed and investigated with respect to their effectiveness in improving medication adherence. Unfortunately, these studies showed inconsistent and disappointing results [1–4]. Interventions that seem most effective often employ multiple components by combining elements of existing interventions [1]. This complicates the evaluation of the impact of these interventions, which in turn challenges the interpretation of research outcomes. Insight into the delivery of (multicomponent) interventions is necessary to gain a better understanding of the underlying reasons that cause interventions to succeed or fail.

One way to gain insight into the way an intervention is delivered is through the assessment of implementation fidelity, defined as the degree to which an intervention is implemented as intended by the developers [5, 6]. Implementation fidelity can act as a potential mediator of the relationship between the intervention and the intended outcome [7]. Several studies have shown that interventions with high fidelity had better outcomes, when compared to interventions with lower fidelity [5, 8]. Although measuring implementation fidelity helps researchers to understand whether a lack of effectiveness is due to poor implementation or inadequacies in the design of the intervention. The systematic assessment of implementation fidelity of intervention studies has often been neglected [5, 7, 9, 10]. However, in recent years the importance to perform this assessment as a base for effective clinical guideline implementation in community pharmacies has increasingly been recognized [11–13].

The Cardiovascular medication non-Adherence Tailored Intervention (CATI) study, a pragmatic randomised controlled trial (RCT) with 9 months of follow up, has recently been carried out to investigate the effectiveness of a patient-tailored, pharmacist-led intervention programme on self-reported medication adherence in 20 community pharmacies [14]. In short, 170 patients (45–75 years) using antihypertensive medication and who were non-adherent, according to both pharmacy dispensing data (refill non-adherence) and a self-report questionnaire (intake non-adherence), participated. Patients randomised to the intervention group received two consultations with a pharmacist. During the first consultation, participants’ barriers to adhere to medication were identified, and tailored information and advice was provided to overcome these barriers. After 2–3 months, a follow-up consultation was planned to discuss participants’ experiences with the initially provided information and advice. Participants in the control group received usual care according to the guidelines of the Royal Dutch Pharmacists Association [15]. This care consists of reviewing and dispensing of the prescribed medication, providing instructions on its use as well as information about intended effects and possible side effects during first and second dispensing. The CATI study showed no significant effects on self-reported medication adherence or other secondary outcomes [16]. The process evaluation of the CATI study might clarify whether the ineffectiveness of the intervention was due to poor implementation or inadequacies in the design of the intervention.

## Aim of the study

The present study aims to evaluate the implementation fidelity and potential moderating factors that might have influenced the implementation of a patient-tailored, community pharmacist-led intervention programme to enhance adherence to antihypertensive medication.

### Ethics approval

All procedures performed in studies involving human participants were in accordance with the ethical standards of the institutional and/or national research committee and with the 1964 Helsinki declaration and its later amendments or comparable ethical standards. The Medical Ethics Committee of the VU University Medical Center Amsterdam approved this study (no. 2015/219). Informed consent was obtained from all individual participants included in this study.

## Method

### Study design

**Fig. 1:**
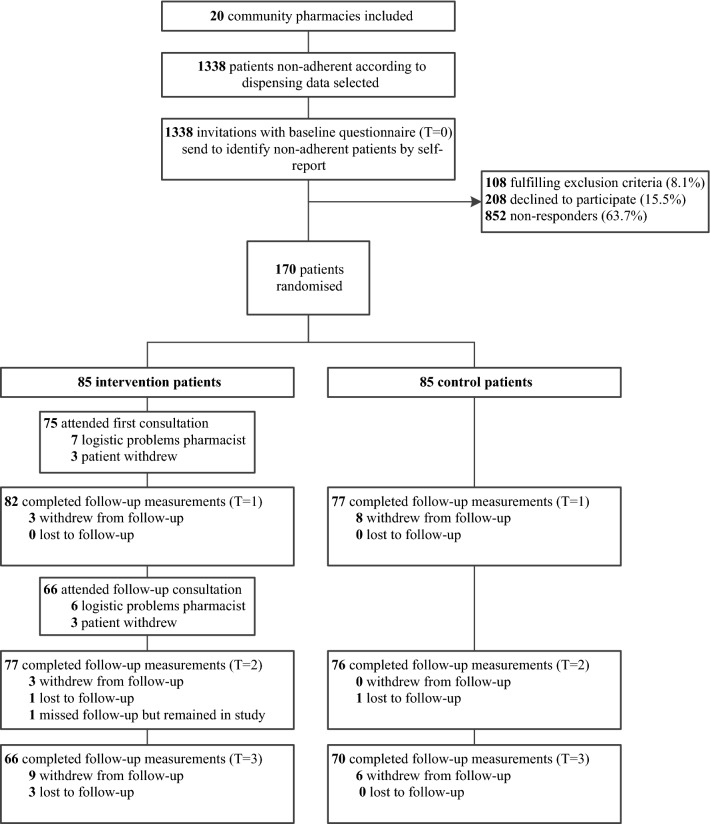
Flow chart of the CATI study participants

The study design and methods of the CATI study have been described in more details elsewhere [14]. A flow chart of the study is presented in Fig. 1. After trial completion, both quantitative and qualitative data were used to evaluate the implementation fidelity of the intervention programme. Quantitative data were collected both during and after the intervention by using pharmacy records, questionnaires and process documents. For the qualitative data collection, we conducted semi-structured interviews with all participating pharmacists after completion of the study.

### Conceptual framework for implementation fidelity

**Fig. 2:**
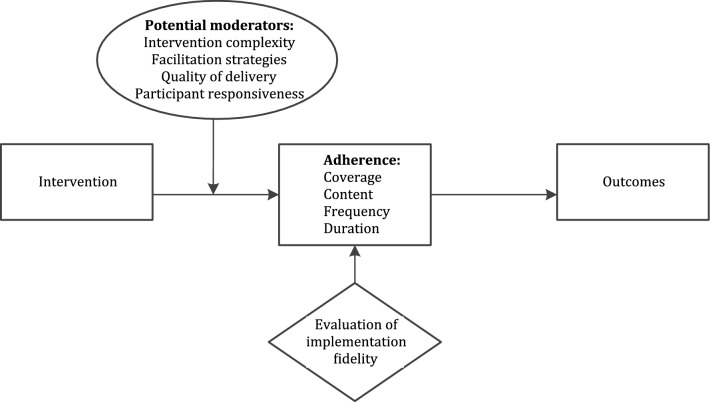
The modified version of the Conceptual Framework for Implementation Fidelity of Carroll et al. [7]

To assess the implementation fidelity, a modified version of the conceptual framework proposed by Carroll et al. [7] was used (Fig. 2). In this conceptual framework, the main element of implementation fidelity is the measurement of *adherence* to the intervention, defined as the degree to which the intervention has been delivered as intended by developers. Adherence can be operationalised by the following categories: coverage, content, frequency and duration. Coverage refers to the proportion of participants exposed to the intervention as intended. Content refers to what extent components of the intervention were delivered as planned. Frequency and duration refer to the delivery of the intervention at the intended intensity. In this framework, potential moderating factors, which might influence the implementation process and as such the level of fidelity, must be considered. The four potential moderating factors are: intervention complexity, facilitation strategies, quality of delivery and participant responsiveness [7]. Briefly, intervention complexity refers to both the comprehensiveness of the intervention protocol and the complexity of the intervention itself. Facilitation strategies refer to strategies such as the provision of manuals and training. Quality of delivery concerns whether an intervention is delivered in a way appropriate to achieving what was intended. Finally, participant responsiveness considers the extent of commitment to the intervention by both participants receiving the intervention and health care providers responsible for delivering it.

For the evaluation of the implementation fidelity of the intervention programme, four key intervention components were identified by the researchers. These key intervention components included:

A. First consultation—barrier identification
  During the first consultation, participants’ barriers to adhere to medication should be identified by means of the Quick Barrier Scan (QBS), which consists of 11 questions representing various barriers, such as lack of knowledge, forgetfulness and side effects. In addition, one open-ended question explores potential other barriers according to the participant [14].
B. First consultation—information & advice
  Based upon the barriers identified, at least one out of five corresponding intervention modules (IM) should be selected from the Tailored Intervention Guide (TIG) [14]. This guide provides an overview of intervention recommendations that pharmacists should use to inform and advise participants to overcome the identified barriers. Information should be provided about hypertension, the use of and need for antihypertensive medication and living a healthy lifestyle. Moreover, participants’ representations of hypertension and its treatment should be discussed. Interventions recommendations, for instance, include changing medication regime in accordance with the general practitioner, using intake-supporting tools (pill box, medication alarm), registering for pharmacy intake-supporting services or discussing negative medication-related beliefs with the pharmacist.
C. First consultation—written summary
  At the end of the first consultation, the participant should be handed a written summary of the consultation, including both the information and recommendations provided.
D. Follow-up consultation
  During the follow-up consultation, participants’ implementation of and experiences with the information and recommendations provided previously should be discussed according to protocol instructions.

Specific research questions per key intervention component for the different elements of the conceptual framework have been presented in Tables 1 and 2. A subjective rating was independently performed by two researchers (DL, MG) in order to provide a score to each research question evaluating the implementation fidelity (Table 1). This was assessed by rating the extent to which the different aspects of the intervention were carried out as planned (low, moderate, high). The percentage of agreements between the two researchers was 67%. The scores were discussed until consensus was reached.

**Table 1:** Overview per key intervention component of research questions, data sources and outcomes for the evaluation of adherence

Key intervention components	Research questions	Data source	Outcomes	Rating
*Coverage*				
In general	What proportion of selected patients were invited to participate?	I	In order to include enough patients and to reach the pre-calculated sample size, seven additional pharmacies were recruited, resulting in a total of 20 pharmacies. In total, 1338 patients from 20 pharmacies were selected with the SFK database search and invited to participate. The intended amount of 75 patients to select per pharmacy was achieved in 13 pharmacies. In other pharmacies the amount ranged from 26 to 74 patients, mainly depending on the size of the pharmacy	Moderate
	How was the selection of patients perceived by pharmacists?	IV	A considerable number of pharmacists indicated that for a few participants, the refill non-adherence was explained by missing pharmacy dispensing data. Pharmacists also indicated that the degree of participants’ intake non-adherence assessed by a self-report questionnaire was limited	Moderate
	Was the selection procedure performed according to the study protocol?	I	The method to select patients according to the pharmacy dispensing data was adequately performed. When the information on switching drugs and hospital stays was available to the researcher could easily verify gaps in the dispensing records. The self-reported questionnaire on medication adherence was administered adequately to the patients	High
	How was the eligibility of participants perceived by pharmacists?	IV	Some pharmacists were not sure whether certain participants were eligible for the intervention, since they did not experience substantial difficulty with their medicine intake or only used one or a few medicines. Moreover, pharmacists perceived that the degree of adherence was already quite high among selected some participants	Moderate
	What proportion of invited patients did not respond, was not eligible or declined participation? And why?	I	Of the 1338 patients that were invited, 852 (63.7%) did not respond, 108 (8.1%) did not meet the inclusion criteria and 208 (15.5%) declined to participate. Reasons to decline participation were no interest, no time, or not considered useful	Moderate
	What proportion of invited patients participated? And why? How was drop-out?	I, V	170 patients (12.7%) were eligible and willing to participate, and 85 patients were randomised to the intervention group. For 53 out of 69 complete cases (76.8%), the reason to participate was finding it important to contribute to scientific research. During follow-up, 29 participants (17.1%) withdrew and five participants (2.9%) were lost to follow-up	Moderate
*Content*				
A. First consultation—barrier identification B. First consultation—information & advice C. First consultation—written summary D. Follow-up consultation	To what extent were the different components of the first consultation delivered as planned?	III, IV	For 62 out of the 75 participants (82.7%) who attended the first consultation, at least one barrier was identified by means of the QBS. For 55 out of the 62 participants (88.7%) with an identified barrier, the correct corresponding IM (including tailored information and recommendations) was selected. For most incorrect selections, the pharmacist identified a barrier with the QBS, but they did not perceive it as a specific reason for medication non-adherence and therefore decided to only provide information and no specific recommendations. For 47 out of the 75 participants (62.7%), a written summary was made at the end of the first consultation including the discussed information and advice. The content of the summaries varied; however, the content was rated as clear and extensive by the researchers for the majority of written summaries	High
	To what extent was the follow-up consultation delivered as planned?	II, IV	For the 66 participants that attended the follow-up consultation, their experiences with and implementation of information and advice in the prior period were discussed. However, objective data concerning the implementation of intervention recommendations by participants have not fully been reported. Nevertheless, it can be concluded that for at least 35 of the 66 participants (53.0%), there was an improvement or change in adherence-related beliefs or behaviour. For instance, multiple participants reported becoming more aware of the necessity of antihypertensive medication and reported less forgetting due to the use of pill boxes, reminder systems and making changes to daily habits. In addition, participants seemed more actively involved with their medicines, and in a few cases, healthy lifestyle changes were made with regard to smoking, eating and exercise behaviour	Moderate
*Frequency and duration*				
A. First consultation—barrier identification B. First consultation—information & advice C. First consultation—written summary D. Follow-up consultation	How many first and follow-up consultations were performed?	I	The first consultation was completed by 75 out of 85 intervention participants (88.2%). Ten consultations were not performed due to withdrawal of participants (*n *= 3) or logistic and time management problems of pharmacists (*n *= 7). There were 66 follow-up consultations. If a participant did not attend the first consultation, a follow-up consultation was not applicable. The number of first consultations per pharmacy varied between two and six, with an average of four first consultations per pharmacy	High
	How many times were the different components of the first consultation performed?	III, IV	On average, two barriers were identified per participant. The most often identified barriers were related to forgetfulness, side effects and perceived necessity of medication use. For 13 participants (17.3%), no clear barrier was identified with the QBS. The most frequently discussed recommendations were related to supportive medication-intake tools. For 47 out of 75 participants (62.7%), a written summary was made. Pharmacists’ most frequently mentioned reason for not making a summary was not seeing the need for it, because the amount of information was limited and the provided recommendations were simple to remember	High
	What was the average consultation time of the first and follow-up consultation?	II	The average time of the first consultation was 36 min (range: 15 to 85 min). The average time of the follow-up consultation was 20 min (range: 5 to 45 min)	Moderate
	How many days were there between both consultations?	I	On average, there were 94 days (range: 20 to 158 days) between the first and follow-up consultation	High

I, researcher administration; II, pharmacist administration; III, data from intervention materials; IV, semi-structured interviews with pharmacists; V, participant evaluation questionnaire

*IM*, intervention module, *QBS* Quick Barrier Scan

**Table 2:** Overview per key intervention component of research questions, data sources and outcomes for evaluation of moderating factors

Key intervention components	Research questions	Data source	Outcomes^a,b^
*Intervention complexity*			
A. First consultation—barrier identification B. First consultation—information & advice C. First consultation—written summary	How detailed was the protocol description of the first consultation?	IV	The majority of pharmacists evaluated the protocol description as clear and informative. A few pharmacists indicated that the protocol description was too detailed, extensive and time-consuming to fully read in advance of the study
	How complex were the different components of the first consultation?	IV	Most pharmacists positively evaluated the intervention materials, especially the use of the flow chart. Herewith, pharmacists were able to identify barriers and were easily guided to the corresponding intervention module. A few pharmacists found it difficult to switch between asking the standardized questions of the QBS and having a normal conversation. For participants were no clear barrier could have been identified or for which the degree of non-adherence was quite limited, pharmacists indicated that it was difficult to properly use the intervention materials, since for these participants providing information and advice seemed not necessary
D. Follow-up consultation	How detailed was the protocol description of the follow-up consultation?	I	During the study, a few pharmacists indicated that the protocol description for the follow-up consultation was unclear. Therefore, pharmacists received additional protocol instructions prior to the execution of the follow-up consultation, including which questions to ask and what information to document
	How complex was the follow-up consultation?	IV	The pharmacists that made a written summary indicated that it was an easy way to start the follow-up consultation. Almost all pharmacists indicated that a follow-up is necessary for these kind of interventions, since it is important to monitor patients over time
*Facilitation strategies*			
A. First consultation—barrier identification B. First consultation—information & advice C. First consultation—written summary D. Follow-up consultation	What were strategies to support the implementation of the first and follow-up consultation?	I	*One*-*day training session*: The majority of pharmacists (65%) followed the training session prior to the study, including an introduction in medication adherence, instructions on the study protocol and intervention materials and they practiced with patient-pharmacist role-playing. *Structured protocol*: at the start of the study, pharmacists received a detailed manual with information on study procedures and intervention materials. *Intermediate instructions*: pharmacists also received intermediate written instructions. *Intensive* m*onitoring*: the researcher had extensive contact with pharmacists in order to monitor progress and meeting deadlines and to provide feedback if necessary. *Incentives*: pharmacists received financial compensation for every intervention participant that attended the consultations. Participants did not receive incentives
	How were these strategies perceived by the pharmacists?	I, IV	All pharmacists rated the *one*-*day training session* as useful and sufficient in terms of content and practical applicability. Only three pharmacists indicated that they wanted to practice their communication skills even more extensively. Based upon the pre-post knowledge and competences test developed by the researchers, eight of the 13 pharmacists who attended the training session (61.5%) improved their knowledge, and three pharmacists (23.1%) remained the same. Almost all pharmacists (90%) reported that their competences related to communicating with patients about medication adherence were enhanced. The majority of pharmacists evaluated the *detailed protocol* as informative and useful and indicated that it was helpful that there were multiple contact moments with the researcher to *monitor* the progress. Pharmacists appreciated the relatively small financial compensation as an incentive for the execution of the intervention
*Quality of delivery*			
A. First consultation—barrier identification B. First consultation—information & advice C. First consultation—written summary D. Follow-up consultation	How was the quality of the different components of the first consultation evaluated by pharmacists?	IV	Almost all pharmacists rated the intervention materials as of good quality. They indicated that by using the QBS, they were able to identify barriers in most cases and that by means of the flow chart, the corresponding IM and recommendations were easy to select. Some pharmacists did not see the need for making a written summary at the end of the first consultation
	How was the quality of the first consultation evaluated by participants?	V	Almost all participants (59 out of 63 complete cases) agreed that the consultations were pleasant. For 39 out of 66 complete cases (59.1%) of the participants, the information and advice were helpful, and even 13 out of 66 complete cases (19.7%) of the participants indicated it was very helpful. Participants rated the first and follow-up consultation with eight points on average on a satisfaction scale from 0 to 10. Certain participants appreciated that pharmacists took enough time to discuss their medication and were convinced that the patient-provider relationship can improve by means of these consultations
	How was the quality of the follow-up consultation evaluated by pharmacists?	IV	Most pharmacists indicated that the follow-up consultations were necessary to follow-up on participants’ implementation of the provided recommendations and also to monitor the intake behaviour in the prior period. When no clear barriers were identified during the first consultation, they indicated that a follow-up consultation was not needed and difficult to execute. Pharmacists indicated improvements of participants’ engagement to and appreciation of the pharmacy due to the personal consultations
	How was the quality of the follow-up consultation evaluated by participants?	V	For 31 out of 63 complete cases (49.2%) of the participants, the follow-up consultation was of added value, and the majority of participants (61 out of 69 complete cases) indicated they would recommend the consultations to others. Most participants (52 out of 69 complete cases) would again ask for help from a pharmacist in case of medication-related problems in the future, whereas 14 participants would rather ask the general practitioner
*Participant responsiveness*			
In general	To what extent were participants in need for help at the start of the study?	V	Only a small number of participants (20 out of 69 complete cases) indicated that they were in considerable need for help, and only a few participants (5 out of 69 complete cases) indicated that difficulties with medicine use had a substantial negative influence on their daily life
	How engaged and satisfied were participants with the intervention?	I, IV, V	The attendance rate of the first and follow-up consultation was 88.2% and 78.8%, respectively. Most missed consultations were because of time management problems of pharmacists, rather than due to lack of participant interest. According to pharmacists, the willingness of participants to engage varied. Some participants were receptive for advice and willing to seriously address the problem, while others were willing to listen but did not want to make any effort to change, did not find it necessary or useful or did not find the time to make changes. For a few participants, it became clear that they only participated to contribute to scientific research or for doing the pharmacist a favour. Most participants (52 out of 66 complete cases) reported that the consultations helped them to better cope with difficulties. In addition, the majority of the participants (48 out of 68 complete cases) rated the provided information and advice by the pharmacist as useful. About one-third of participants indicated that their knowledge was increased (28 out of 69 complete cases) and their medicine intake was improved (26 out of 69 complete cases) due to both consultations
	How engaged and satisfied were pharmacists with the intervention?	I, IV	The majority of pharmacists were well engaged with the intervention; however, for a few pharmacists, implementation of the intervention was difficult, and frequent monitoring was needed. In one pharmacy, the pharmacist devoted the execution of the intervention to a pharmacy technician. The missed consultations were mainly because of logistic and time management problems of pharmacists. The majority of pharmacists indicated that the intervention was useful for supporting patients with adherence problems. Moreover, most pharmacists would like to perform these kind of consultations with their patients in the future. Some pharmacists indicated that it was difficult to deliver the intervention in a proper manner to participants that seemed not eligible for the intervention

I, researcher administration; II, pharmacist administration; III, data from intervention materials; IV, semi-structured interviews with pharmacists; V, participant evaluation questionnaire

*IM*, intervention module, *QBS*, Quick Barrier Scan

^a^Since a pharmacy technician executed the intervention programme in one pharmacy, data reported about pharmacists concerns 19 pharmacists and one pharmacy technician

^b^Data from multiple participants is missing, the number of complete cases are written in brackets

### Data collection

To answer the specific research questions, the following data sources, numbered with roman numerals (I–V), were used.

#### Data from researcher

The researcher kept administrative records (I) on the logistics and process of the implementation of the intervention programme. The researcher recorded data on the flow of participants throughout the study.

#### Data from pharmacists

Pharmacists filled out an administration form (II) for each participant, including a description of the pharmacists’ impression of both the first and follow-up consultation and data on time investment (in minutes) per consultation. In addition, during the first consultation, the pharmacists used intervention materials (III), from which data concerning identified barriers and provided information and recommendations were retrieved. Semi-structured interviews with all participating pharmacists (IV) were conducted at the end of the study. The following topics were included: eligibility of included participants, impression of the first and follow-up consultations, usability of intervention materials, feasibility of implementing the intervention programme in daily practice and advantages and disadvantages of the intervention programme.

#### Data from participants

Participants from the intervention group filled out an evaluation questionnaire (V) in order to assess their reasons for participation and need for help prior to the study and their experiences and satisfaction with the different components of the intervention programme. This questionnaire was self-composed by the researchers and consisted of several closed-ended questions rated on a Likert scale (agree, neutral, disagree). In addition, using a small number of open-ended questions, participants were invited to describe their impression of the first and follow-up consultations in more detail.

### Data analyses

Quantitative data were presented as frequencies with percentages. For the quantitative data that were collected by the evaluation questionnaire, only results of participants with complete data were reported. For the qualitative data collection, semi-structured interviews were audio taped and transcribed. The transcripts were analysed using the Framework approach [17]. Two independent researchers (DL, MG) coded the transcripts based on the interview topic list. Subsequently, the coded transcripts were arranged to broader themes. Differences were discussed until consensus was reached. Qualitative data were analysed using Atlas.ti software version 7 (GmbH, Berlin).

## Results

Details on the outcomes per key intervention component for each aspect of the conceptual framework are presented in Tables 1 and 2.

### Adherence

#### Coverage

In order to include a sufficient number of participants and reach the pre-calculated sample size, seven additional pharmacies were recruited, resulting in a total of 20 participating pharmacies. Of 1338 selected patients from 20 community pharmacies, 170 patients (12.7%) were eligible to participate, of which 85 were randomised to the intervention group and 85 to the control group. According to the participants’ evaluation questionnaires, the most reported reason to participate in this study was contributing to scientific research. Patients were included if they were non-adherent, according to both pharmacy dispensing data (refill non-adherence) and a self-report questionnaire (intake non-adherence). In the semi-structured interviews some pharmacists indicated that the non-adherence classification according to refill data for some participants might have been caused by missing dispensing data, which resulted in misclassification of these participants. In addition, pharmacists perceived the degree of intake non-adherence in some participants so minimal that they assumed limited potential for improvements in medication adherence. Pharmacists also indicated that some participants did not seem eligible for the intervention, because of the limited number of prescribed medicines and the lack of structural difficulties with medicine intake. When comparing the implementation of the selection procedure with the study protocol [14], both methods to select patients were performed adequately and as intended. However the ability of these methods to identify genuinely non-adherent patients was considered insufficient.

#### Content

Based on the filled out intervention materials, the identification of participants’ barriers to adhere to medication by means of the QBS was delivered as planned. For nearly all participants, one or more barriers were identified. Subsequently, pharmacists provided information and advice using the correct corresponding intervention module of the TIG to almost all participants. In the semi-structured interviews, pharmacists indicated that for certain participants they did not perceive the identified barrier as being causative for the medication non-adherence and therefore decided to provide only general information about hypertension. Providing a written summary for participants to take home was carried out as planned for almost two-thirds of the participants. The discussion of participants’ experiences with the information and advice provided at the first consultation seems to have been implemented as planned, based on the descriptions of the follow-up consultation in pharmacists’ administration forms. However, data concerning which recommendations were actually implemented by participants has not fully been reported objectively. Nevertheless, pharmacists indicated that about half of the participants appeared to have acted upon the advice provided at the first consultation, as they displayed an improvement in adherence-related beliefs or behaviour.

#### Frequency and duration

For 75 out of 85 intervention participants, a first consultation was performed. Reasons for not performing this consultation were mainly related to logistic and time management problems of pharmacists. For 66 participants, a follow-up consultation was performed. At the first consultation, an average of two barriers were identified per participant (Table 3). No barrier was identified for 13 participants. The most frequently discussed recommendations were related to the use of supportive medication-intake tools, which corresponds to data of the second intervention module (Table 4). The average durations of the first and follow-up consultations were 36 and 20 min, respectively. On average 94 days passed between the first and follow-up consultations. A written summary was made for 47 of 85 participants. Pharmacists’ most frequently mentioned reason for not making a written summary was not seeing the necessity.

**Table 3:** Frequencies and percentages of identified barriers according to Quick Barrier Scan and the corresponding intervention module for intervention participants (N = 62)

Quick Barrier Scan	N (%)^a^	Corresponding IM
Do you believe you have insufficient knowledge about your disease or medicines?	22 (35.5)	IM1
Do you forget to take your medicines on regular days?	29 (46.8)	IM2
Do you forget to take your medicines on irregular days?	20 (32.3)	IM2
Do you experience side effects of your medicines?	23 (37.1)	IM3
Do you experience anxiety about developing side effects?	4 (6.5)	IM3
Do you have difficulties with medicine intake due to a complex intake schedule?	8 (12.9)	IM4
Do you have difficulties with opening packages or swallowing pills?	6 (9.7)	IM4
Do you experience negative beliefs about medicines in general?	11 (17.7)	IM5
Do you believe that the use of your prescribed medicines is not necessary?	20 (32.3)	IM5
Do you believe that your prescribed medicines are not effective or that the disadvantages of your medicines outweigh the advantages?	9 (14.5)	IM5
Do you not quite so much still enjoy the things you used to enjoy?	6 (9.7)	IM5

IM1, Providing Information; IM2, Providing Supportive Tools; IM3, Dealing with Side Effects; IM4, Overcoming Practical Problems; IM5, Diminishing Negative Beliefs

*IM* intervention module

For 13 out of 75 participants that attended the first consultation, no clear barrier was identified (for these cases IM1 should have been selected)

^a^Multiple barriers could have been identified for each participant, therefore the total amount exceeds 100%

**Table 4:** Frequencies of discussed recommendations per intervention module based upon the Tailored Intervention Guide

Intervention module	Recommendations for participants to overcome barriers	N^a,b^
IM1	Visit preselected informative websites on hypertension or adequate medicine intake	8
IM1	Read provided information leaflets on hypertension or adequate medicine intake	13
IM1	Get additional information or support from other health care providers	6
IM2	Try to connect medicine intake to daily habits, e.g. brushing teeth, coffee break	25
IM2	Ask for support with medicine intake from friends or family	6
IM2	Try out the adjusted schedule of medicine intake	4
IM2	Purchase a pill box to organise and store multiple medicines	12
IM2	Use a reminder system to prevent forgetting	11
IM2	Download a smartphone application as a reminder or supportive tool	15
IM2	Register for the pharmacy dispensing service: pill packaging	4
IM2	Register for the pharmacy dispensing service: repeat dispensing	21
IM2	Permit the pharmacist to contact GP for medication review if desired	3
IM3	Try to weigh out disadvantages of side effects with advantages as discussed with pharmacist	17
IM3	Permit the pharmacist to contact GP for medication review if desired	8
IM4	Try out the adjusted schedule of medicine intake	3
IM4	Try out the instructions on how to open packages or how to press through pills	1
IM5	Try to weigh out disadvantages of medicines in general with advantages as discussed with pharmacist	2
IM5	Try to weigh out disadvantages of prescribed medicines with advantages as discussed with pharmacist	3
IM5	Permit the pharmacist to contact GP to discuss potential depressive symptoms	1

IM1, Providing Information; IM2, Providing Supportive Tools; IM3, Dealing with Side Effects; IM4, Overcoming Practical Problems; IM5, Diminishing Negative Beliefs

*IM* intervention module, *GP* general practitioner

^a^Data of the discussed recommendations from 23 out of 75 participants is missing

^b^Multiple recommendations were provided per participant, therefore the total amount exceeds the number of participants

### Moderating factors

#### Intervention complexity

At the semi-structured interviews, the majority of pharmacists evaluated the protocol description of the first consultation as clear and informative. Some pharmacists indicated that it was extensive and too time-consuming. Nearly all pharmacists indicated that the intervention materials were clear and easy to use. After identifying a barrier, pharmacists found themselves easily guided to the corresponding intervention module to provide participants with tailored information and advice. For participants that seemed less eligible for the intervention, pharmacists indicated that it was difficult to use the intervention materials according to protocol. Some pharmacists found the protocol description of the follow-up consultation somewhat ambiguous. Therefore, all pharmacists received additional protocol instructions during the study.

#### Facilitation strategies

A 1-day training session, a structured protocol, intermediate instructions, intensive monitoring and financial incentives were provided to facilitate the implementation of both consultations by pharmacists. Seven pharmacists did not attend the 1-day training session, of which four were unable to attend that specific day and three had not yet been included in the study. The pharmacists who did not attend the training session received individually received additional instructions at the start of the study. Attending pharmacists rated the training session useful as a preparation for study participation. According to the pre-post assessment, the majority found to have improved their knowledge and competences at the training [14]. Three pharmacists desired more communication skills exercises. Pharmacists considered the structured protocol and intensive monitoring supportive to implement the intervention programme. Moreover, pharmacists appreciated the financial compensation for their willingness to participate.

#### Quality of delivery

According to pharmacists, the intervention materials available for the first consultation were of high quality. At the semi-structured, pharmacists indicated that they could easily identify barriers for adherence for most participants and inform and advise them accordingly. Almost one-third of pharmacists did not see the need for making a written summary at the end of the first consultation. Pharmacists indicated that the follow-up consultation was important for most participants to monitor the feasibility of the advice provided previously. As assessed by the evaluation questionnaire, most participants rated the consultations with the pharmacists as pleasant. Participants also indicated appreciation and satisfaction for the personal attention and provided support from pharmacists. Half of the participants indicated the follow-up consultations were of added value, and nearly all participants would recommend participating in these consultations to others patients.

#### Participant responsiveness

As assessed by the evaluation questionnaire, about one-third of participants indicated to be in considerable need for help at the start of the study. A few participants indicated that difficulties with the use of their medication had a substantial negative influence on their daily life. Despite a limited proportion of participants indicated a need for help, the attendance rates of the first and follow-up consultations were quite high. In the evaluation questionnaire, the majority of participants indicated that the information and advice was helpful and allowed them to better cope with difficulties regarding medication intake. With regard to the responsiveness of participating pharmacists, the majority of the pharmacists were well engaged to the intervention. However, two pharmacists had difficulty with implementation of the intervention and required frequent monitoring. One pharmacist devoted the execution of the intervention to a pharmacy technician. At the semi-structured interviews, most pharmacists indicated that the intervention would be useful for supporting patients with serious adherence problems in daily practice and that they would like to perform similar consultations with their patients in the future.

#### Feasibility of intervention programme

Additional information regarding the feasibility of the CATI intervention programme, beyond the scope of the conceptual framework for implementation fidelity, was obtained from the semi-structured interviews conducted with the pharmacists. Most pharmacists indicated that they believed the intervention programme to be feasible in daily practice. However, they emphasised that it would be better to focus on smaller groups of patients. In addition, the majority of pharmacists indicated the benefit of asking for the assistance of a pharmacy technician for logistics and administration of the programme, and some pharmacists believed that a pharmacy technician would be able to execute the programme, following appropriate communication and consulting training. A few pharmacists indicated that the current design of the intervention programme was not feasible in practice, since the consultations are very time consuming. Suggested changes were to replace the face-to-face consultation with a consultation per telephone. From the viewpoint of logistics, this would save time and allow for more participants to be reached. Pharmacists also indicated that they preferred the intervention materials to be integrated in their pharmacy information systems which would allow to intervene with system-identified non-adherent patients when they visit the pharmacy for a refill. Some pharmacists also suggested that it would be beneficial to expand clinical medication reviews with adherence-enhancing consultations.

## Discussion

The present study aimed to evaluate the implementation fidelity of a patient-tailored, community pharmacist-led intervention programme to enhance adherence to antihypertensive medication. According to the rating of the researchers, the implementation fidelity was moderate to high for all key intervention components, meaning that these components were mostly carried out as planned. However, the method to select patients was considered insufficient, and pharmacists questioned the eligibility for the intervention of some participants. Moreover, we cannot rule out selection bias, since large numbers of patients did not respond or declined to participate.

The implementation fidelity of the four key intervention components was evaluated. The first consultation was carried out as planned. The identification of participants’ barriers to adhere to medication as prescribed and the provision of tailored information and advice were well implemented for most participants. However, one-third of the participants was not given a written summary at the end of the first consultation. Despite protocol instructions, some pharmacists did not see the necessity of the written summary. Therefore, it appears that the instructions were not sufficiently clear or that the importance of this step had not sufficiently emphasised. At last, the limited eligibility of some participants made it difficult to use the intervention materials properly, thereby hindering its implementation for a small proportion of participants.

The training of pharmacists, intensive monitoring during the study and the structured and easy-to-use intervention materials were found to have facilitated the implementation of the intervention. Pharmacists also appreciated the financial incentive, however, they indicated that a large-scale implementation in daily practice would require higher reimbursement due to the time required to conduct the intervention.

Since the intervention programme was mostly developed based on our previous research, comparable intervention studies in the literature are scarce. Two previous studies with quite comparable interventions reported on the effectiveness of the intervention but did not perform a process evaluation [18, 19]. This reflects the shortcoming in the current literature, in which process evaluations of multicomponent interventions are often neglected [7, 9, 10]. Therefore, an adequate comparison with the current literature is not feasible.

### Strengths and limitations

A strength of our process evaluation is that it provides insight into the delivery of an intervention, including whether the lack of effectiveness of the intervention was due to poor implementation [9]. Moreover, the use of a conceptual framework in order to systematically evaluate the implementation fidelity has been recommended in the literature [7, 20]. Finally, by using several data sources, we were able to obtain a wide variety of information. The study of the process evaluation also had its limitations. First, a subjective rating was used by the researchers to value the implementation fidelity. However, it should be recognised that applying an objective rating in this type of process evaluation seems unfeasible. Second, some aspects of the conceptual framework for implementation fidelity have not been assessed extensively. For instance, assessment of the quality of delivering the intervention was limited. Audio recordings of the consultations might have provided more insight into the character and quality of the communication between pharmacist and participant. Moreover, interviews with participants might have provided more insight into participant responsiveness. It is recommended that the complexity of the intervention is evaluated by an external group of researchers; however, it was not feasible to obtain an external assessment [20].

### Implementation in daily practice

The implementation fidelity was evaluated alongside a pragmatic randomised controlled trial, which does not fully resemble daily practice. The researcher selected patients in each pharmacy, sent invitations for participation and reviewed the filled-out self-reported questionnaires for eligible patients. Preferably by using the pharmacy information system, community pharmacists or pharmacy technicians must be instructed on how to select eligible patients for adherence-enhancing interventions in order to allow the successful implementation of this multicomponent intervention in daily practice. Moreover, more insight is needed into which methods can be used to properly select non-adherent patients as such. Performing the consultations by a community pharmacist appears to be feasible in practice. However, with respect to logistics and administration the involvement of a pharmacy technician would be helpful. A recent review concluded that pharmacy technicians are a valuable asset to community pharmacists in the process of implementing and operation of adherence programmes [21]. The amount of time spent on preparing and performing the consultations limits the feasibility. An effort should be made to reduce consultation time while maintaining the quality of the consultations. Changes suggested by pharmacists that could reduce consultation time were integrating the intervention materials in the pharmacy information system and replacing the face-to-face consultation with a consultation per telephone. Studies have shown positive effects of telephonic interventions on medication adherence, confirming the efficacy of this method [22, 23]. Particularly with respect to the time required to adequately perform the intervention, reimbursement is required for pharmacists performing the consultations with patients. Pharmacists in the Netherlands are reimbursed for conducting clinical medication reviews with patients. It might therefore be reasonable to make adherence-enhancing consultations an optional component of a clinical medication review.

In addition to the changes suggested to allow successful implementation of the present intervention programme in daily practice, some general factors relevant to fidelity of pharmaceutical care implementation in community pharmacies should be considered. Alongside strong methodological designs of intervention studies, including a tailored approach and a theoretical framework, it is important to consider factors like adaptability, context and climate, logistics support by staff and sustainability of the community pharmacy setting [11, 13, 24].

## Conclusion

In this process evaluation, nearly all key intervention components were carried out as planned. Therefore, the absence of effectiveness of the intervention programme on self-reported medication adherence cannot be explained by a poor implementation of the intervention. However, the possibility of a selection bias and the questionable eligibility of certain participants, mainly due to a rather low degree of intake non-adherence, appeared to have resulted in a limited potential for improvement of medication adherence. Extensive communication skills training, easy-to-use and system-integrated intervention materials, appropriate time and resource allocation and genuinely eligible patients appear to be necessary elements for successfully implementing adherence-enhancing interventions in daily practice.
